# Practical application of digital therapeutics in people with mood disorders

**DOI:** 10.1097/YCO.0000000000000906

**Published:** 2023-11-01

**Authors:** Laura Orsolini, Giulio Longo, Umberto Volpe

**Affiliations:** Unit of Clinical Psychiatry, Department of Neurosciences/DIMSC, Polytechnic University of Marche, Ancona, Italy

**Keywords:** bipolar disorder, depression, digital mental health, digital therapeutics, mood disorders

## Abstract

Digital therapeutics (DTx) offer evidence-based digitally-delivered high quality standards applications and/or softwares in the prevention, management and treatment of several medical conditions, including mood disorders. Nowadays, there are only three DTx officially approved by the Food and Drug Administration for mental conditions and there are still very few DTx developed in the context of mood disorders. The current comprehensive overview aims at providing a summary of currently published studies on DTx clinical applications in major depressive disorder (MDD), depressive symptomatology and bipolar disorder (BD), by using PubMed/MEDLINE and Scopus databases. Fifteen studies have been selected (10 on DTx in depressive symptomatology and/or MDD; 4 on BD; 1 on MDD and BD). Literature on DTx in mood disorders is still lacking, being mostly constituted by feasibility and acceptability rather than efficacy/effectiveness outcomes, particularly in BD. More studies focused on MDD compared to BD. Most DTx on MDD have been developed based on cognitive behaviour therapy interventions while on BD are based on psychoeducation. All studies assessing symptom severity improvement pre- vs. postinterventions demonstrated a significant postintervention improvement. Therefore, despite the preliminary encouraging results of studies here retrieved, their methodology is still too heterogeneous to allow comparisons and the generalizability of their findings. Further studies are warranted, in more larger samples involving multiple sites, including measures of both specific symptom effects as well as acceptability, feasibility and effectiveness in the real-world settings.

## INTRODUCTION

According to the World Health Organization (WHO), about one in eight people nowadays suffer from mental disorders, mostly represented by mood (particularly depression) and anxiety disorders, further increased due to the coronavirus disease 2019 (COVID-19) pandemic [1]. In WHO's Comprehensive Mental Health Action Plan 2013–2030 it is planned to boost mental health promotion and prevention strategies, as well as to strengthen the mental health information systems as well as digital opportunities in mental disorders [1]. Indeed, digital psychiatry may offer different prevention, management, and treatment interventions for a broad spectrum of physical, mental, and behavioural conditions [2]. In the field of mental health, many digitally-delivered interventions have been developed for delivering effective and feasible treatments for several mental conditions, including mood disorders [2]. Indeed, few digital mental health interventions are effectively supported by scientific and clinical evidence, such as the digital therapeutics (DTx). 

**Box 1:**
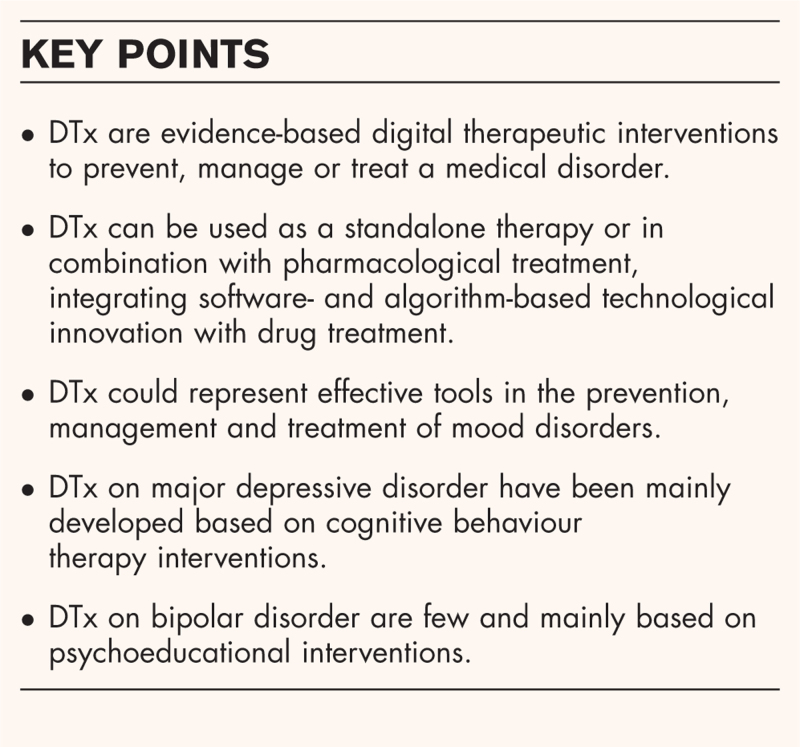
no caption available

The term DTx was firstly introduced by Joseph Kvedar in 1995, to identify all evidence-based therapeutic interventions driven by software to prevent, manage or treat a medical disorder or disease [3]. DTx uses digital implements such as mobile devices, apps, virtual reality, the Internet of Things (IoT), wearable sensors and so forth to spur patients’ behavioural changes. DTx can be used as a standalone therapy or in combination with pharmacological treatment, integrating software- and algorithm-based technological innovation with drug treatment [2,3]. DTx are classified as Medical Devices and, hence, they need to be specifically developed with high scientific quality and validated standards through randomized controlled clinical trials (RCTs), as for drug-based therapies [3]. More specifically, DTx are classified in two categories: SaMDs (i.e., ‘software as a medical device’) and SiMDs (i.e., ‘software in a medical device’). SaMDs indicate a software that functions as an actual medical device, while SiMDs is a software that is included within a medical device for treating a specific medical condition [4]. When a SaMD or SiMD is embedded within a smartphone it is called a ‘mobile medical app’ (MMA) [5].

Furthermore, DTx by definition are also supported by clinical evidence coming from real-world outcomes, as well as approved by regulatory agencies [such as the Food and Drug Administration (FDA)], prescribed by physicians and (only in some countries) reimbursed by national health systems (NHS) and/or private insurances [6]. At the European level, there are not still specific legal regulations on DTx assessment or to guarantee their safety and the integrity of data collected. Currently the European Medicines Agency (EMA) and the European Commission (EU) are starting to explore DTx as therapeutic solutions. At national level, the new German Digital Healthcare Act (DiGA) regulates specific requirements for the use of DTx (i.e., quality, security and data protection) and France is moving towards a similar act [6]. In the UK, the National Institute for Health and Care Excellence (NICE) set up a working group under the guidance of the NHS to support regulatory boards in identifying which types of evidence are most relevant to the assessment of DTx [6]. To date, three DTx products have been recommended by NICE (but not yet approved): ‘Deprexis’ (an interactive medical device for unipolar depression); ‘Space from Depression’ (an online programme for the treatment of depression); and BDD-NET (an online programme for treating moderate-to-severe body dysmorphic disorder) [7–9] (Table 1). While in the USA, FDA has an active precertification program on DTx in place since 2017. However, FDA currently approved only three DTx in the field of mental health: reSET (a computerized behavioural therapy device for substance use disorders); Somryst (a computerized behavioural therapy device for treating chronic insomnia); and Endeavour (a serious game for young patients with ADHD) [10^▪^–12^▪^] (Table 2).

**Table 1:** Characteristics of NICE-recommended DTxs for use in the field of mood disorders

Deprexit	Space for depression	BDD-NET
• Online CBT programme • Available in 9 languages • 10 weekly modules including mindfulness and acceptance, and problem solving • Adult patients with unipolar depression or depressive mood disorders • Aims: acquiring effective coping strategies to manage depressive symptomatology and/or depression	• online CBT programme • 5 weekly modules including psychoeducational content (delivered in multiple formats to facilitate acquisition of knowledge and promote usability), interactive tools and activities to reinforce learning and to encourage reflection and implementation of new skills. • Adult patients with depression • Aims: acquiring effective coping strategies to manage depressive symptomatology and/or depression • Weekly reviews by trained therapists provide guidance, feedback and motivation to users	• Online CBT programme • 12-week treatment program based on current psychological models of body dysmorphic disorder including psychoeducation, functional analysis, cognitive restructuring, exposure and response prevention, and relapse prevention modules. • Patient with moderate-to-severe body dysmorphic disorder • A dedicated therapist provides active guidance and feedback throughout the entire process

CBT, cognitive behaviour therapy; DTx, digital therapeutics.

**Table 2:** Characteristics of FDA-approved DTxs in the field of mental health

reSET	Somryst	EndeavorRx
• Cognitive behavioural therapy • 12 weeks (90 days) prescription-only treatment • Patients with substance use disorder from 18 years old • Aims: increase abstinence during treatment and increase retention in the outpatient treatment program • Lessons: identifying situations and triggers that make substance use more likely, avoiding substance use, coping with thoughts about substance use, recognizing negative thinking and identifying techniques to move to positive thinking, making decisions about substance use, taking responsibility for choices made and evaluating the consequences of those choices. • Every lesson is delivered primarily through text, sometimes videos, animations and graphics. • After most therapy lessons, patients undergo a questionnaire to evaluate learning.	• Cognitive behavioural therapy • 9 weeks (63 days) prescription-only treatment • Patients with chronic insomnia from 22 years old • Aims: improving patients’ insomnia symptoms • The therapy consists in text, video, animation and graphics.	• 25 min per day for 5 days per week • Patient with Attention Deficit Hyperactivity Disorder (ADHD) from 8–12 years old • EndeavorRx has to be considered for use as part of a therapeutic program • EndeavorRx is built on Akili's proprietary, patented, technology platform and uses adaptive algorithms (also known as Selective Stimulus Management Engine, SSME) to deliver stimuli that are designed to engage the patient in a manner that improves their attention function. In a closed-loop system, the adaptive SSME algorithms automatically adjust the difficulty level for a personalized treatment experience that is tailored to the needs of each individual patient. • Delivered through a video game experience which leverages art, music, storytelling, and reward cycles to keep patients engaged. • The basic program inputs are steering, which is accomplished by using the internal accelerometer to measure the degree to which the mobile device is tilted, and tapping, which is accomplished using the touch screen to measure correct and incorrect targeting. The basic outputs are a visual display of the game progression along with audio, which is accomplished by using the internal high-resolution display and internal speaker.

CBT, cognitive behaviour therapy; DTx, digital therapeutics.

DTxs could represent effective tools in the prevention, management and treatment of mood disorders [13]. However, given the limited availability of DTx officially approved by abovementioned regulatory bodies, there is the need to provide a comprehensive overview on currently available studies aiming at investigating the practical applications of DTx in the context of mood disorders, including major depressive disorder (MDD) and bipolar disorder (BD).

## METHODS

A comprehensive literature review has been carried out here in order to better deepen the practical applications of DTx in mood disorders, including peripartum depression. Studies were identified searching the electronic databases MEDLINE/PubMed and Scopus. A combined search strategy of free text terms and exploded MESH headings for the topics of Digital Therapeutics (DTx) and Mood Disorders as following: (((*Digital Therapeutics*[Title/ Abstract]) OR (*DTx*[Title/ Abstract])) AND ((*Mood Disorder*[Title/Abstract]) OR (*Depression*[Title/ Abstract]) OR (*Bipolar Disorder*[Title/ Abstract]))), without time restrictions, through September 29, 2023. In addition, further studies were retrieved from reference listing of relevant articles and consultation with experts in the field and or manual search. We limited the search to only English-written studies. The following exclusion criteria have been applied: not human studies; studies on DTx without data on its application in mood disorders; studies on digital mental health but not specifically addressed to DTx; studies on DTx applied in mood disorders in comorbidity with physical and/or other mental conditions; studies discussing only protocols without clinical data; studies on already NICE recommended DTx for mood disorders. Identified studies were independently reviewed for eligibility by two authors (G.L. and L.O.) in a two-step-based process; a first screening was performed based on title and abstract while full texts were retrieved for the second screening. At both stages, disagreements by reviewers were resolved by consensus. Data were extracted by two authors (G.L. and L.O.) and disagreement was resolved by a third author (U.V.) using an ad-hoc developed data extraction spreadsheet. With the initial set of keywords, by integrating all databases, some 1,135 studies were identified. After screening and selection by using inclusion criteria, only 20 articles were selected. Of these 15 relevant studies were finally included. Table 3 summarizes the main findings of studies here retrieved. Findings have been discussed according to different diagnostic groups (i.e., studies concerning MDD, BD or both).

**Table 3:** Characteristics of the included studies

Study	Product	Intervention	Targeted population	Study participants	Study type	Design	Outcome measure
**Studies on DTx in MDD and Depressive Symptomatology**							
Fitzpatrick et *et al.*, 2017 (USA)	Woebot	CBT	Young adults aged 18–28 years-old with depressive and anxiety symptomatology	*n* = 70 randomized to Woebot (*n* = 34) and self-guide on depression (*n* = 36)	Feasibility, acceptability, and preliminary efficacy evaluation	RCT	Efficacy in symptom severity pre vs. post Use and acceptability Qualitative feedbacks from participants
Iacoviello *et al.*, 2018 (USA)	n/a	EFMT	Adult with un-medicated MDD	*n* = 51 randomized to EFMT (*n* = 28) or sham-control (*n* = 23)	Efficacy and acceptability evaluation	RCT (randomization to EFMT and sham-control group involving working memory training)	MDD symptom severity pre vs. post Working memory effects pre vs. post Symptom-level analysis pre vs. post Acceptability and perceived helpfulness
Goldin *et al.*, 2019 (Finland)	Ascend	CBT and mindfulness meditation exercises	Adults with depressive symptoms	*n* = 117	Feasibility evaluation	Longitudinal Observational Study	Feasibility (drop-out rates, daily practice, weekly group chat use) Symptom severity improvement pre vs. post-
Bower *et al.*, 2020 (UK)	ADvisor	Psychoeducation	Adults taking antidepressants for more than 1 year for a first episode or 2 years for a subsequent episode; discontinued antidepressants, or in the process of tapering without current persisting symptoms of depression and without suicide ideation or history of suicide attempts and without any comorbid psychiatric disorders	*n* = 15	Intervention development	Evidence and person-based mixed-methods (quantitative and qualitative) study	Patient's views on barriers and facilitators to withdrawal, the role of healthcare professionals in supporting withdrawal attempts and elements of a proposed intervention to support withdrawal
Venkatesan *et al.*, 2020 (USA)	Vida Health's app	CBT	Adults with mild-to-moderate depression and/or anxiety symptomatology	*n* = 323	Effect evaluation	Retrospective Study	Long-term outcomes Intervention engagement Symptom severity improvement pre vs. post-
Gould *et al.*, 2021 (USA)	Meru Health app	CBT and mindfulness	Middle-aged and older adults (aged≥40) with severe depressive symptomatology	*n* = 20	Feasibility and effect evaluation	Single-arm pilot RCT	Symptom severity improvement pre vs. post Participants’ experiences Acceptability Feasibility
Tang *et al.*, 2022 (USA)	MamaLift Plus	CBT and IPT	Women >18 years old with mild-to-moderate PPD	*n* = 14	Acceptability and usability evaluation	Single-arm, mixed-methods (quantitative and qualitative) study	Acceptability Usability Participants’ experiences Feasibility
Suharwardy *et al.*, 2023 (USA)	Woebot	CBT and Interpersonal psychotherapy (IPT)	Women >18 years old at-risk for PPD within 72 hh after their delivery and with access to smartphone	*n* = 192 randomized to chatbot or TAU	Feasibility and effect evaluation	unblinded, single-center RCT	Acceptability Feasibility Symptom severity improvement pre vs. post-
Kulikov *et al.*, 2023 (USA)	Spark	CBT	Adolescent (aged 13–18) with depressive symptomatology	*n* = 60 randomized to Spark (*n* = 35) and active control (*n* = 25)	Feasibility and acceptability evaluation	RCT	Symptom severity improvement pre vs. post Usability Engagement Participants’ safety
Gual-Montolio *et al.*, 2023 (Spain)	“My EMI, Emotional Well being’ app	’T	Adults with moderate-to-severe anxiety or depressive symptomatology and with a computer or a mobile phone Internet access	*n* = 30	Feasibility evaluation	Single-group, open trial	Usability and acceptability Satisfaction Adherence to app and treatment (attrition and dropout rates) Feasibility Symptom severity improvement pre vs. post-
**Studies on DTx in BD**							
Depp *et al.*, 2010 (USA)	PRISM	Psychoeducation	Adults with BD	*n* = 20	Feasibility and effect evaluation	Pilot study	Acceptability Participants’ feedbacks
Dodd *et al.*, 2017 (UK)	ERPonline	Psychoeducation	Adults with BD (type-1 and type-2) not in acute phase, but at risk of relapse (≥3 previous episodes, ≥1 in the preceding 2 years) and with access to Internet	*n* = 19	Feasibility, engagement and usability evaluation	Single-arm, qualitative study	Intervention satisfaction (barriers and facilitators to both recruitment and retention; engagement with ERPonline website; reflections on using online support)
Lobban *et al.*, 2017 (UK)	ERPonline	Psychoeducation	Adults with BD (type-1 and type-2) not in acute phase, but at risk of relapse (≥3 previous episodes, ≥1 in the preceding 2 years) and with access to Internet	*n* = 96	Feasibility and Acceptability evaluation	Single-blind RCT with nested qualitative study comparing ERPonline plus TAU vs. waitlist control (with delayed access to ERPonline plus TAU)	Feasibility (recruitment and retention rates, data completion, direct feedback from participants) Effectiveness Acceptability (usage, number and type of adverse events, detailed feedback from participants about their experiences)
Jonathan *et al.*, 2021 (USA)	LiveWell	Psychoeducation	Adults with BD (type-1 and type-2) with ≥2 acute mood episodes within the preceding 2 years	*n* = 12	Feasibility and usability evaluation	Evidence and person-based mixed-methods (quantitative and qualitative) study	Usability Participants’ experiences Feasibility
**Studies on DTx in MDD and BD**							
Cho *et al.*, 2020 (Korea)	CRM app	Psychoeducation	Adults with MDD, BD (type-1 and type-2)	*n* = 73 randomized to CRM (*n* = 14) and non-CRM group (*n* = 59)	Effect evaluation	Case-Control Study with 12-months follow-up	Symptom severity improvement pre vs. post Recurrence rate pre vs. post in both groups Effectiveness of feedback intervention on behavioural changes

CBT, cognitive-behaviour therapy; CRM, Circadian Rhythm for Mood; EFMT, Emotional Faces Memory Task; ERP, Enhanced Relapse Prevention; IPT, interpersonal therapy; MDD, major depressive disorder; n/a, not applicable; PPD, postpartum depression; RCT, randomized clinical trials; TAU, treatment-as-usual.

## RESULTS

### Depression

Iacoviello *et al.*[14] developed a cognitive-emotional training intervention for MDD, named ‘Emotional Faces Memory Task’ (EFMT), consisting of exposing patients to photos of faces in sequence on a computer. Postintervention results demonstrated a significant improvement in emotional recognition faces among patients as well as an effective improvement of working memory.

Goldin *et al.*[15] developed the ‘Ascend program’ characterized by 8 module interventions delivered via a mobile phone-based app in 8 weeks. This program consists of cognitive behaviour therapy (CBT) activities, mindfulness and behavioural activation therapy. Modules are delivered in the following order: ‘Introduction to mindfulness’, ‘Low mood and motivation’, ‘Self-compassion’, ‘Managing worry’, Overcoming thinking traps’, ‘Rethinking your life values’, ‘Being aware of your relationships’, and ‘Relapse prevention’. The aim is to provide patients with tools for managing depressive episodes. Findings demonstrated a significant long-term reduction of depressive symptoms over 4-weeks postintervention.

Bower *et al.*[16] developed ‘ADvisors’, a digital intervention prototype, specifically aimed to assist patients during discontinuation of antidepressant therapy. This intervention is designed to educate patients about medication taken, especially by providing information regarding side effects and discontinuation symptoms. These types of activities would also allow for greater control by physicians over the patient's health.

Venkatesan *et al.*[6] conducted a study to investigate the effectiveness of ‘Vida Health's’ app-based CBT program. Specifically, the app allows patients to connect with a therapist, who provides a set of activities and goals based on the progress of therapy each week. The results demonstrated a significant reduction in depressive symptomatology, which maintained stability over time.

Gould *et al.*[17] investigated the efficacy of the ‘Meru Health’ app addressed to adults and older adults with depressive symptomatology. The app works by delivering CBT and mindfulness-based therapy by trained psychotherapists on a daily basis through informative materials and videos. It also allows patients to ask for an asynchronous conversation with the therapist and a group discussion. Findings highlighted a good feasibility and a significant reduction in depressive and anxiety symptomatology.

Tang *et al.*[18] developed the app ‘MamaLift’ addressed to postpartum depression, consisting of a self-guided intervention that can be delivered independently or with the support of a therapist. Each day it is proposed to the patient a self-guided program based on CBT, IPT, dialectical behaviour therapy (DBT) and behavioural activation therapy (BAT) through videos, audios and text formats. Findings found a high level of satisfaction, feasibility and usability by the patients.

Kulikov *et al.*[19] developed the ‘Spark’ app consisting of a self-guided 5-weeks CBT, specifically addressed to adolescents aged 13–18 patients. Outcomes reported a high level of engagement and satisfaction by patients, as well as a significant reduction in depressive symptoms.

Gual-Montolio *et al.*[20] described the effects of ‘My EMI’, and app to promote emotional well being in a sample of adults with emotional disorders, including depressive symptomatology. The intervention is based on Measurement-Based Care (MBC), which consists of regular monitoring of patients, periodic feedback to the therapist (or both therapist and patient), and adaptation of the intervention based on these feedbacks.

Chatbots are software applications designed to replicate human conversations, enabling users to interact with digital devices as if they were engaging with a genuine person [21]. Fitzpatrick *et al.*[22] used a chatbot, Woebot, in young adult patients with depressive symptoms. Suharwardy *et al.*[23] conducted studies on the use of Woebot in the context of postpartum depression. This intervention equips patients with tools from CBT and interpersonal therapy (IPT) to manage mood and anxiety. Both studies reported a significant depressive symptomatology reduction, although Suharwardy *et al.*[23] did not include only women with confirmed diagnosis of postpartum depression.

### Bipolar disorder

Depp *et al.*[24] conducted a study using a mobile intervention, called ‘PRISM’, which provides BD patients with information from the BD psychoeducational intervention in a self-managed approach, without the intervention of a therapist. Through a series of mood questions, the app also creates a mood chart to monitor mood trends. The results showed a reduction in depressive symptoms but not of manic symptoms.

Dodd *et al.*[25] and Lobban *et al.*[26] conducted two studies based on ‘Enhanced Relapse Prevention’ (ERPonline) addressed to BD subjects. It is a self-directed web-based intervention that allows people to create a model of their mood fluctuations, allowing them to recognize and manage triggers of new episodes and develop new effective coping strategies. The intervention has been described by patients as accessible, relevant and straightforward [25,26].

Jonathan *et al.*[27] and Dopke *et al.*[29] developed a self-management ‘LiveWell’ app for BD patients, which provides information about disorder and learns specific skills to manage mood fluctuations and recurrences. At the same time, it allows the development of a Wellness plan to reduce the risk of relapse and the management of signs and symptoms in acute phases. It also allows monitoring of treatment adherence, sleep duration, and wellness [27,28].

### Depression and bipolar disorder

Cho *et al.*[29] conducted a study on patients with major depressive disorder and bipolar disorder constituted by multiple digital interventions. Each patient was asked to fill out a daily eMood chart and use a wearable daily activity tracker. An app named ‘Circadian Rhythm for Mood’ collects all passive and active data to predict mood trends and identify specific subject's relapse variables. ‘Circadian Rhythm for Mood’ demonstrated to significantly reduce the number and duration of depressive and manic episodes, promoting also the development of healthy protective behaviours.

## DISCUSSION

Overall, there is an urgent public health need for more evidence-based, high quality, more portable and accessible, as well as more effective and clinically validated DTx in the real-world settings for MDD and BD. Web-based interventions, particularly DTx, may offer the potential to broaden access, reduce waiting times, delivery costs and stigma as well as improve quality through standardized and clinically validated delivery. Current retrieved literature on DTx applications in the field of mood disorders appear still lacking, being mostly constituted by feasibility and acceptability studies rather than efficacy and/or effectiveness studies. There are more studies specifically addressed to MDD, even though mostly recruited subjects with depressive symptomatology, not necessarily with a MDD diagnosis (Table 3); being more represented by adult subjects with only one study addressed to young adults [22], only one study recruiting adolescents [19] and one study included older adults [17]. Two studies were carried out on women at-risk for postpartum depression (PPD) [18,23]. Moreover, most studies on MDD were conducted in the USA and in a single-site and recruited a small sample size (with less than 100 recruited subjects) (Table 3). Most DTx on MDD or depressive symptomatology have been developed based on CBT interventions, with one study based on EFMT [14] and the two only studies on PPD which integrate both CBT and IPT [18,23]. While only four are the published studies evaluating DTx on BD samples, with mostly recruiting a relatively small sample size (≤20 recruited subjects) and based on feasibility and/or acceptability outcomes as well as psychoeducational-based interventions (Table 3). Most of the studies on BD were conducted in the USA or UK and on adult subjects. The only study recruiting both MDD and BD individuals was conducted in Korea and investigated the efficacy and effectiveness of DTx in both samples [29]. Overall, all studies assessing symptom severity improvement pre- vs. postinterventions (both addressed to MDD or BD individuals) demonstrated a significant postintervention improvement, even though only in one study it was conducted also a follow-up evaluation over the time to investigate whether their improvements were long-term maintained [29].

Therefore, there are several limitations to be acknowledged in the current review. Firstly, most studies recruited small sample sizes, not sex- and/or -age homogeneous groups, with heterogeneous methodology regarding inclusion criteria and assessment tools, which may limit the generalizability of their findings. Secondly, most studies tested the only feasibility and level of acceptability of DTx without posing as the primary outcome the clinical efficacy and/or effectiveness of the interventions on MDD and/or BD subjects. Thirdly, most studies have been carried out as RCT without evaluating their feasibility in a ‘real-world’ setting or in a large sample of more heterogeneous individuals. Fourthly, most studies did not evaluate the attrition rate (‘not-attrition bias’) and mostly were single-site studies, conducted in the USA or UK. Furthermore, most studies did not evaluate follow-up data to evaluate the sustainability of results over the time.

Furthermore, despite the potential and encouraging results of DTx studies here retrieved, their methodology is still too heterogeneous to allow comparisons and the generalizability of their findings. Finally, further studies are warranted, in more larger samples involving multiple sites, including measures of both specific symptom effects as well as acceptability, feasibility and effectiveness in the real-world settings. Moreover, more studies should be conducted to investigate DTx in MDD and BD by recruiting different age-groups (i.e., adolescents vs. young adults vs. adults vs. elderly) in order to compare whether it may differ from the findings based on the different target population. Furthermore, studies assessing depressive symptomatology should be implemented and verified in the MDD population in order to evaluate their efficacy, effectiveness as well as feasibility and acceptability may differ according to the symptom severity and diagnosis. Finally, further studies should be carried out in order to develop and implement more DTx interventions focussed on CBT, IPT and Interpersonal and Social Rhythm Therapy (IPSRT) on BD population.

## Acknowledgements

*Author contributions: L.O. and G.L. designed the study. L.O. and G.L. collected data. L.O. and G.L. wrote the first draft of the manuscript. U.V. supervised the entire work and revised the first and subsequent drafts of the manuscript.*

*Author Agreement: Our manuscript has been approved by all authors.*

### Financial support and sponsorship

*None.*

### Conflicts of interest

*There are no conflicts of interest.*

## REFERENCES AND RECOMMENDED READING

Papers of particular interest, published within the annual period of review, have been highlighted as:

▪ of special interest
▪▪ of outstanding interest
